# Social contagion of pain and fear results in opposite social behaviors in rodents: meta- analysis of experimental studies

**DOI:** 10.3389/fnbeh.2024.1478456

**Published:** 2024-10-29

**Authors:** Rui Du, Yang Yu, Xiao-Liang Wang, Guofang Lu, Jun Chen

**Affiliations:** ^1^Institute for Biomedical Sciences of Pain, Tangdu Hospital, Fourth Military Medical University, Xi'an, China; ^2^State Key Laboratory of Holistic Integrative Management of Gastrointestinal Cancers and National Clinical Research Center for Digestive Diseases, Xijing Hospital of Digestive Diseases, Fourth Military Medical University, Xi'an, China; ^3^Sanhang Institute for Brain Science and Technology, Northwestern Polytechnical University, Xi'an, China

**Keywords:** empathy, fear, pain, emotional contagion, emotional valence, helping behavior, three-chamber test, dyadic social interaction paradigm

## Abstract

**Introduction:**

The study aimed to explore the key factors influencing emotional valence in rodents, focusing on the critical elements that distinguish the contagion processes of fear and pain.

**Methods:**

Through a systematic review and meta-analysis, we examined behavioral outcomes of rodents exposed to painful or fearful catastrophes to see whether they are prosocial or antisocial through three-chamber test and dyadic social interaction paradigm.

**Results:**

Fear contagion, particularly when witnessed, leads to social avoidance behavior, unaffected by sex difference but more pronounced with age. In contrast, pain contagion promotes social approach and caring/helping behaviors.

**Discussion:**

The present study demonstrates that the emotional valence induced by pain contagion is quite different from fear contagion and this difference may result in different motivations and social behaviors, namely, social contagion of pain is likely to be more associated with prosocial behaviors, however, social contagion of fear is likely to be more associated with antisocial behaviors.

**Systematic Review Registration:**

PROSPERO (CRD42024566326).

## 1 Introduction

Psychologically, Elaine Hatfield explains that emotional contagion is a straightforward and automatic process where observing another person's emotional state can cause one to “catch” that emotion and display similar emotional and physiological responses (Hatfield et al., 1993). Frans de Waal defines emotional contagion as the alignment of emotional states between individuals, while empathy comprises three capabilities: (a) being influenced by and sharing other's emotional state, (b) understanding the cause of the other's state, and (c) adopting the other's perspective (de Waal, 2008). From an evolutionary point of view, empathy can also be defined as an evolutionary trait associated with prosocial reciprocity, altruism and morality, encompassing the ability to feel, recognize, understand and share others' emotional states (Chen, 2018).

Emotional contagion and empathy are vital for social animals, allowing them to quickly and automatically connect with the emotional states of others. This connection is crucial for regulating social interactions, coordinating activities, and fostering cooperation (de Waal, 2008). With the neural basis for empathy established, it functions not only in caregiving but also within broader social contexts. For example, observing the distress of adult conspecifics can trigger consoling behaviors (de Waal and Preston, 2017; Preston and de Waal, 2017). Emotional contagion is common in the animal kingdom, particularly among highly social species like primates and rodents. This emotional alignment strengthens bonds and cooperative behaviors within groups, enhancing survival and reproductive success (Chartrand and Bargh, 1999). It allows individuals to quickly respond to the emotional states of group members, adjusting behavior to meet environmental and social demands. This is critical in complex social interactions, aiding in maintaining social order and harmony (Sharpe and Cherry, 2003). Additionally, emotional contagion and empathy have adaptive functions for group survival. Observing stressed conspecifics may signal threats, prompting others to respond appropriately (Panksepp and Lahvis, 2011). Thus, emotional contagion and empathy are essential not only for individual well-being but also for the overall adaptation and evolution of social animals.

Fear contagion and pain contagion show distinct behavioral differences. Fear contagion occurs when an individual perceives other's emotional expressions related to danger or threat, prompting escape behaviors (de Waal, 2008). Observational fear learning involves rodents forming fear memories by observing freezing response of conspecifics to electrical foot shock. The social transmission of fear requires the observer to perceive and share the demonstrator's emotional state (Keum and Shin, 2019; Kim et al., 2021). This behavior spans preventive actions before contact to freezing upon contact, eventually leading to a fight-or-flight response when a threat is imminent (Abend, 2023; Vieira and Olsson, 2024). In contrast, pain contagion involves an individual experiencing increased pain responses upon perceiving other's pain (de Waal, 2008). This phenomenon is rooted in empathetic responses, activating neural circuits associated with personal pain experiences, driving prosocial behavior, and motivating helping actions (Vieira and Olsson, 2024). Therefore, pain contagion not only underscores empathy for others' suffering but also highlights the motivation for prosocial responses.

Using fear contagion as a model for empathy research has potential misunderstandings and limitations. It is unclear if the dynamics and kinematics of fearful movements differ quantitatively and qualitatively from neutral actions or those expressing other emotions, which could lead to an oversight of other emotional contagion mechanisms, affecting the comprehensiveness and accuracy of the research (de Gelder et al., 2004). Avoidance behaviors induced by fear contagion include delayed feeding, shortened feeding duration, and extended time to complete tasks. These behaviors primarily aim to reduce personal fear rather than to demonstrate true empathetic responses. This differs from empathy's core trait, which involves understanding and emotionally engaging with and sharing other's emotional state, resulting in approach and helping behaviors, not just self-protection (Gachomba et al., 2024). Historically, monkeys have refrained from pulling a chain that would provide them food but shock a companion, eliciting a pain response. However, this behavior might not necessarily indicate concern for the companion, as it could also be seen as avoiding an aversive side effect (Masserman et al., 1964). In experiments where rats administer shocks to their partners, some rats experience fear through emotional contagion and switch to a non-shocking lever to avoid this negative state. Researchers might misinterpret these actions as altruistic, whereas they may be driven by self-interest (Carrillo et al., 2019). Thus, it is unclear if the observed vicarious social fear in rodents indicates emotional empathy since their vicarious freezing behavior might be emotional contagion without an intrinsic understanding of the distress source (Keum and Shin, 2019).

This study meticulously examined existing studies on fear and pain contagion among laboratory rodents, particularly emphasizing the use of the three-chamber test apparatus to gauge attention and emotional preferences, alongside research on general social behavior and caring or helping behavior. The goal was to delve into the factors that influence emotional valence caused by the complexity of emotional contagion in rodents, identifying the critical elements that differentiate the contagion processes of fear and pain. A clear understanding of the behavioral definitions used in this study was referred to Supplementary Table S1.

## 2 Methods

This systematic review and meta-analysis were registered with PROSPERO (CRD42024566326). We followed the preferred reporting project for systematic reviews and meta-analysis recommendations (Moher et al., 2009; Shamseer et al., 2015).

### 2.1 Information source and search strategy

**Figure 1:**
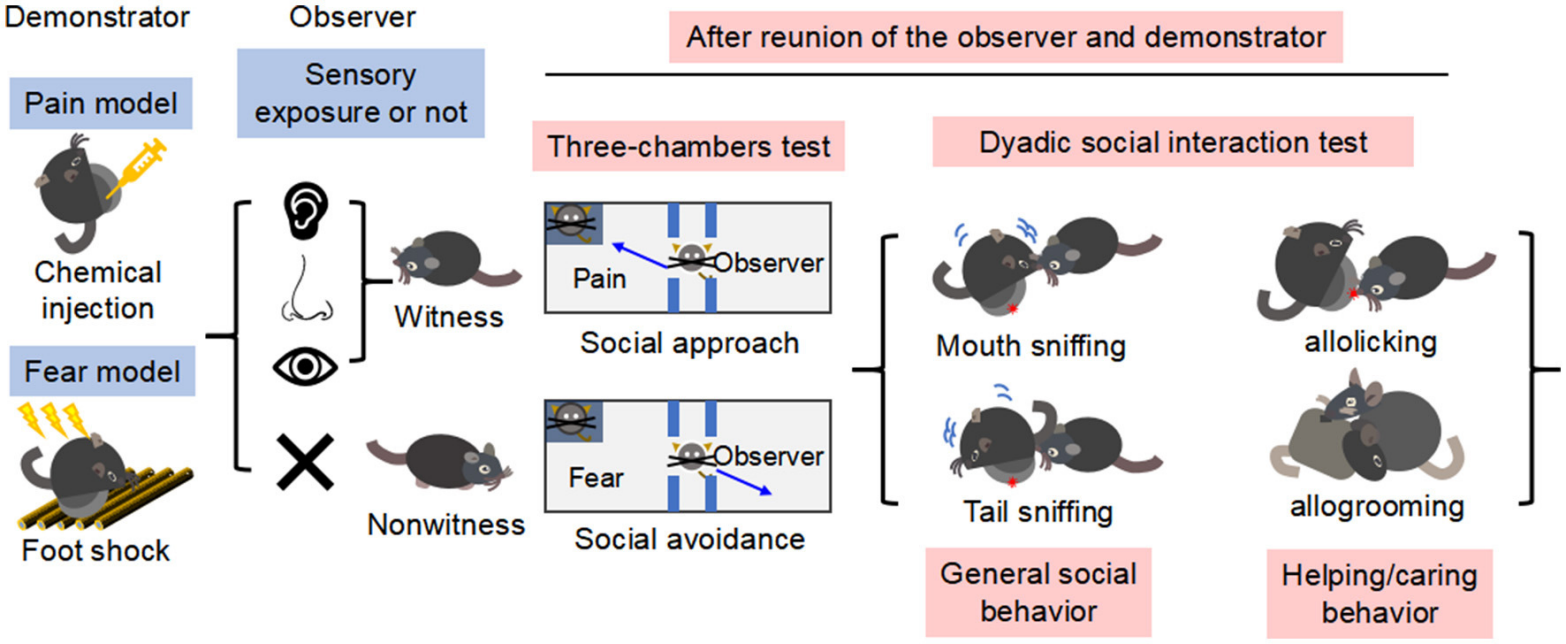
A timeline and experimental protocols of fear and pain contagion models and social behavioral tests discussed in this meta-analysis. As being illustrated, before reunion of the observer and demonstrator for social interaction, sensory modalities were noted as a critical variable in terms of witness (an observer would have been exposed directly to the catastrophic event occurred on the demonstrator) or nonwitness (the observer would have been blind to how the demonstrator was treated). After preparation of pain or fear model, the observers would be subjective to a series of social tests including three-chambers test and dyadic social interaction test to see whether they are prosocial (social approach and more helping/caring) or antisocial (social avoidance and less helping/caring).

This meta-analysis explored two primary inquiries (Figure 1): (1) the magnitude of emotional contagion, focusing on social behaviors such as approach or avoidance, in both male and female rats and mice, and (2) the critical factors influencing emotional contagion in these animals. Emotional contagion was characterized as behavioral reactions prompted by observing others in pain or fear.

We used three databases to search for the relevant literature: PubMed, Medline and Web of Science. For each database, we constructed filters which included the same words [(“empathy” OR “emotional contagion” OR “social transmission” OR “vicarious”) AND (“witness” OR “fear” OR “freezing” OR “approach” OR “avoidance” OR “pain”) AND (“mice” OR “rats”)]. For all three databases, a time and language filter were applied, to only include research articles published in English between 01/01/1969 and 07/19/2024. Outputs from different databases were consolidated into a unified list, from which duplicates were eliminated to curate a final selection of pertinent studies. Each article was scrutinized against our inclusion criteria: (1) Written in English, with manual checks for any that were missed. (2) Utilized rat or mouse models, focusing our analysis on these species due to their common use in research. (3) Presented original empirical findings rather than reviews or opinions. (4) Reported on emotional contagion, specifically social behaviors like approach or avoidance, for effect size analysis.

Our criteria for selecting studies hinged on how demonstrator mice and rats showed social approach behavior, interpreted as the drive to initiate or engage in positive social interactions, or social avoidance behavior, indicative of a tendency to distance from social interactions (Figure 1). These behaviors, grounded in the research of Elliot et al. (2006) for approach and Heuer et al. (2007) for avoidance, served as a basis for analyzing social motivations in response to different stimuli. This tendency might stem from fears such as negative judgment, social anxiety, or perceived threats within social contexts, especially in situations deemed risky. To mitigate selection bias and enhance objectivity in our study selection, we applied a structured approach, using predefined criteria based on the earlier mentioned definitions of social approach and avoidance. This method guided our evaluation of whether the findings from each reviewed article satisfied our inclusion standards. To meet our inclusion criteria, studies had to: (1) analyze behaviors in rats or mice; (2) involve behaviors triggered by emotional cues from another animal of the same species, such as distress signals; (3) encompass both direct (e.g., visible distressed animal) and indirect cues (e.g., distress-related odors or sounds); (4) focus on neutral emotional cues (like social buffering scenarios) often using setups like a three-chambers apparatus for unbiased conspecific interaction decisions.

### 2.2 Data coding and management

A specialized coding sheet was created to document relevant details such as animal characteristics, experiment setup, and risk of bias in quantification. Authors R.D. and Y.Y. thoroughly reviewed and recorded data from each chosen article. For added precision in the data coding, G.L. carried out a secondary check. Considering the extensive data gathered, key parameters for this meta-analysis were selected, including those that might influence emotional contagion and the variables directly measuring it, to compute distinct effect sizes. To ensure consistency in data extraction from all the studies, each variable described in Supplementary Table S2 followed the properties described below.

*Sex*: the coding identified whether studies used male or female animals, categorizing mixed-sex groups as “both.” However, due to a small number of such studies, they were not included in analyses examining the effect of sex.

*Emotion transfer*: the classification of transferred emotions was based on Panksepp's framework (Panksepp, 2011), focusing on two out of seven categories: fear/anxiety and pain/panic. Fear/anxiety was noted when observed animals showed signs like freezing or defecating, indicative of distress. Pain/panic was identified through pain-related responses such as writhing or reactions to mechanical pain, reflecting the witnessing animals' distress.

*Witness/nonwitness:* the definition of witnessing an event typically involves an observer directly seeing a subject (demonstrator) receiving a noxious stimulus, such as an injection of a pain-inducing substance or an electric shock. In this scenario, the observer learns how to respond to a specific stimulus by observing the subject's reactions, including expressions of pain and avoidance behaviors (Helsen et al., 2011). The concept of nonwitness refers to instances where the observer does not directly witness the subject receiving a negative stimulus. This category also encompasses indirect signals, such as odors, which suggest the occurrence of an adverse event without direct visual evidence. The fear paradigm is defined as a dyadic social interaction between the observer and the demonstrator, where the former witnesses or does not witness the latter receiving an electric shock stimulus. The definition of the pain paradigm aligns with that of the fear paradigm in the focus of this meta-analysis.

*Three-chamber test:* the test involves a three-chambers setup widely used in behavioral neuroscience to assess rodents' social preferences, social memory, and solo exploratory behaviors. This configuration typically includes three linked chambers, allowing movement through small doors or passageways.

*General social behavior*: rodent social behavior prominently features sniffing, a key communicative act that enables them to acquire crucial information about their surroundings and fellow creatures through smell. This behavior, characterized by the nose's movement toward another animal's body, particularly the face, neck, and genital regions, serves a vital role in discerning individual identities, sex, reproductive statuses, and social rankings.

*Helping behaviors*: helping behaviors in rodents, such as licking pain sites (allolicking) (Du et al., 2020) or grooming a conspecific's fur (allogrooming) (Geng et al., 2020), serve as manifestations of empathy and attempts to alleviate the discomfort of their counterparts. These behaviors not only reflect social bonds between animals but also demonstrate cognitive and emotional resonance with the feelings of their peers.

In addition to these study characteristics, we assessed the quality and the risks of each screened article. For each study, we indicated whether (1) blinding, (2) randomization, (3) prior calculation of required sample size and (4) declaration of conflict of interest was included in the article (Supplementary Table S3).

### 2.3 Extraction of data of interest and computation of effect sizes

While statistics were reported in most studies, the comparisons often did not directly test emotional contagion. In such cases, descriptive statistics (typically mean and standard error of the mean) were manually extracted from graphical representation of data. In the process of data extraction and effect size calculation, we applied the following equations to compute relevant parameters: Equation 1 calculates the standard deviation (SD) for data presented with the standard error of the mean (SEM). Equation 2 is used to calculate the effect size (ES) for comparisons between two groups, while Equation 3 applies to within-group comparisons. Additionally, we converted effect sizes to correlation coefficients (*r*) using Equation 4, and Equations 5 and 6 are applied to calculate effect sizes based on *t*-values and *F*-values, respectively. Due to the lack of descriptive data in most manuscripts, manual extraction was performed in 86% of the articles scrutinized (*N* = 30). Manual extraction utilizing software-based (WebPlotDigitizer) data extraction was highly accurate confirmed by Lallement (Hernandez-Lallement et al., 2022). When data was presented graphically with the standard error of the mean (SEM), we calculated the standard deviation (SD) using a specific formula to facilitate our analysis and interpretation of the results:


(1) $$\begin{array}{l} SD=SEM\times \sqrt{n} \end{array}$$

Where *n* represents the total number of observations (for example, participants), we utilized the data to calculate the effect size (*r*). For categorical variables undergoing group comparisons, we established a convention assigning positive or negative signs to indicate directionality. The magnitude of deviation from 0 indicated the effect strength. Positivity was ascribed to effect sizes aligning with predefined expectations, facilitating a standardized approach to evaluating and interpreting the results' significance:

1) Witnessing positive emotions in others increases seeking-related behavior, such as locomotion and approach behavior.
2) Compared to the control group, observing the emotional changes in others leads to a greater use of sensory modalities (i.e., auditory, olfactory, and visual) for active exploration.
3) Compared to the control group, observing the emotional changes in others results in an increase in helping behaviors (i.e., allolicking and allogrooming).

In this meta-analysis, we calculated effect sizes as standardized mean differences, adopting methods from Leichsenring (2001) and Lipsey and Wilson (2000). For comparisons involving two distinct groups (such as in a between-subjects design, typically experimental vs. control), we utilized a specific formula to derive the effect size, ensuring our analysis accurately reflects the magnitude of differences observed between groups. This approach allows for a systematic and quantifiable assessment of the study outcomes:


(2) $$\begin{array}{l} ES=\frac{M_{1}\;-\;M_{2}}{\sqrt{\frac{SD_{1}^{2}\;\times \;n_{1}\;+\;SD_{1}^{2}\;\times \;n_{2}}{n_{1}\;+\;n_{2}}}} \end{array}$$

In the calculation, *M*, *SD*, and *n* denote the mean, standard deviation, and sample size for both the experimental group and control group 1 and 2, respectively. For instances involving comparisons within the same group (for example, comparing baseline to a test time point in a within-subjects design), a different calculation method was employed to determine the effect size, enabling a nuanced analysis of changes or impacts within the same set of subjects over time:


(3) $$\begin{array}{l} ES=\frac{M_{t+i}-M_{t}}{\sqrt{SD_{t+i}^{2}+SD_{t}^{2}}} \end{array}$$

In this formulation, *M*_t_ represents the initial mean measurement (typically baseline), *M*_(t+i)_ stands for the subsequent time point measurement, SD_t_ is the standard deviation at the initial measurement, SD_(t+i)_ indicates the standard deviation at the later measurement point, and N denotes the sample size of the group.

To standardize different measures into a unified metric and simplify interpretation, effect sizes were converted into correlation coefficients *r*. This conversion process, rigorously outlined by Hedges and Olkin (1985), involved utilizing a specific formula to translate standardized mean differences into *r*, enhancing the clarity and comparability of the effect size estimations across studies.


(4) $$\begin{array}{l} r=\frac{ES}{\sqrt{ES^{2}+4}} \end{array}$$

When the relevant statistics were provided in the article, effect sizes were computed using the adequate formula:


(5) $$\begin{array}{l} r=\frac{t}{\sqrt{t^{2}+df}} \end{array}$$

Where *t* is the *t* value, and *df* is the degrees of freedom.


(6) $$\begin{array}{l} r=\sqrt{\frac{F}{F+df}} \end{array}$$

Where *F* is the *F*-value, and *df* is the degrees of freedom.

### 2.4 Combining effect sizes and comparisons

We applied the Fisher transformation to normalize effect sizes due to the skewness of *r* values further from 0, following the recommendation of Hedges and Olkin (1985).


(7) $$\begin{array}{l} Z_{r}=0.5\times ln\left[\frac{1+r}{1-r}\right] \end{array}$$

Following the calculation of the effect size *r* through the methods previously described, the $Z_{r}$ value obtained from Fisher's transformation, which normalizes *r*, is reconverted into *r* for easier interpretation, in line with the guidelines suggested by Lipsey and Wilson (2000).

In order to correct for biases caused by low sample size (<20 or 10 in each group, see Nakagawa and Cuthill, 2007), we computed the unbiased $Z_{r}\left(Z_{ru}\right)$ value using the equation proposed by Hedges and Olkin (1985) and Nakagawa and Cuthill (2007):


(8) $$\begin{array}{l} Z_{ru}=Z_{r}\times \left[1-\frac{3}{4\times \left(n_{1}+n_{2}\right)-9}\right] \end{array}$$

Where *n*_1_ and *n*_2_ are sample sizes of two comparison groups, and the $Z_{r}$ is the biased effect size estimated in Equation 7.

Here, we introduce a novel concept, labeling the standardized effect size ($Z_{r}$) as “emotional valence.” Moving forward, a positive $Z_{r}$ value ($Z_{r}>$0) signifies positive emotional valence, illustrated by “social approach (Figures 3–5)” in triadic experiments and an escalation in “allolicking/allogrooming/sniffing behaviors (Figure 6)” during dyadic social interactions. Conversely, negative $Z_{r}$ values ($Z_{r}<$0) are indicative of negative emotional valence, portraying outcomes that starkly contrast with the positive examples provided.

### 2.5 Random effect model

In meta-analytic methodologies, selecting either a fixed effect or a random effects statistical model is essential. The fixed effect model presumes that all effect sizes estimate a uniform effect, while the random effects model accommodates variability in effects across studies. The selection of the model significantly influences the interpretation of summary estimates. To determine the appropriate model, we conducted a heterogeneity test to produce the *Q*-statistic, as outlined in Equation 13. This *Q*-value serves as an indicator of the variation among the effect sizes. The *Q* statistic adheres to the chi-square distribution with *k*-1 degrees of freedom, where *k* represents the total number of effect sizes included. In this meta-analysis, the *Q*-value was highly significant [$\chi_{\left(127\right)}^{2}=689.72,\;p<0.001$], supporting the use of a random effects model. The model posits that the variance of each effect size (*v*_i_, as defined in Equation 9) consists of variance from inherent sampling errors (*v*_o_, outlined in Equations 10, 11) in addition to variance from other sources of randomly distributed variability (*v*_r_, detailed in Equation 11). To estimate these components, we applied Equation 9 through Equation 14, extensively documented by Lipsey and Wilson (2000) and Nakagawa and Cuthill (2007):


(9) $$\begin{array}{lll} \nu_{i} & = & \nu_{0}+\nu_{r} \end{array}$$

(10) $$\begin{array}{lll} \nu_{0} & = & SE^{2} \end{array}$$

(11) $$\begin{array}{lll} SE & = & \frac{1}{\sqrt{n-3}} \end{array}$$

(12) $$\begin{array}{lll} \nu_{r} & = & \frac{Q-\left(k-1\right)}{\Sigma w_{i}-\left(\Sigma w_{i}^{2}/\Sigma w_{i}\right)} \end{array}$$

(13) $$\begin{array}{lll} Q & = & \Sigma w_{i}Z_{ru_{i}}^{2}-\frac{{\left(\Sigma w_{i}Z_{ru_{i}}\right)}^{2}}{\Sigma w_{i}} \end{array}$$

(14) $$\begin{array}{lll} w_{i} & = & \frac{1}{SE^{2}} \end{array}$$

Alongside the *Q* statistic, we computed the *I*^2^ statistic using Equation 15. This metric quantifies the percentage of variance between studies attributable to actual heterogeneity as opposed to random chance, as detailed by Higgins et al. (2003) and Higgins and Thompson (2002):


(15) $$\begin{array}{l} I^{2}=\frac{Q-df}{Q} \end{array}$$

*Q* is determined by Equation 13, where df equals the number of effect sizes minus one. Higher percentage values signify greater heterogeneity. For this meta-analysis, the *I*^2^ = 81.5%, *p* < 0.001, indicating substantial amount of heterogeneity and giving further support for a random model analysis.

For each variable and its different levels, we calculated the mean effect size, 95% confidence intervals (CI) and Z score value using Equations 16–19.


(16) $$\begin{array}{lll} \bar{Z_{ru}} & = & \frac{\Sigma w_{i}Z_{ru_{i}}}{\Sigma w_{i}} \end{array}$$

(17) $$\begin{array}{lll} 95\%\;CI & = & \bar{Z_{ru}}\pm 1.96\times SE_{\bar{Z_{ru}}} \end{array}$$

(18) $$\begin{array}{lll} SE_{\bar{Z_{ru}}} & = & \sqrt{\frac{1}{\Sigma w_{i}}} \end{array}$$

(19) $$\begin{array}{lll} Z & = & \frac{\bar{Z_{ru}}}{SE_{\bar{Z_{ru}}}} \end{array}$$

### 2.6 Statistical analysis

All data are presented as mean ± standard error. Data analysis was performed using SPSS 26.0. The non-parametric Mann–Whitney *U*-test and Spearman test would be utilized if both normality test (Shapiro-Wilk test) and equal variance test (Levene test) for samples failed (Supplementary Tables S6, S7). A *p*-value of <0.05 was considered statistically significant. Graphs were created using Graph Pad Prism 9.4.0. It is worth noting that some studies use the same animals to measure emotional contagion (social approach/avoidance) or moderating factors, as well as their impact on general social/helping behavior. Consequently, more than two non-independent effect sizes were extracted from the same group of animals (*N* = 25 studies). In such cases, the related effect sizes were analyzed separately (see Section 3), ensuring that the effect sizes used in each analysis remained independent (Hernandez-Lallement et al., 2022).

## 3 Results

### 3.1 Main findings

**Figure 2:**
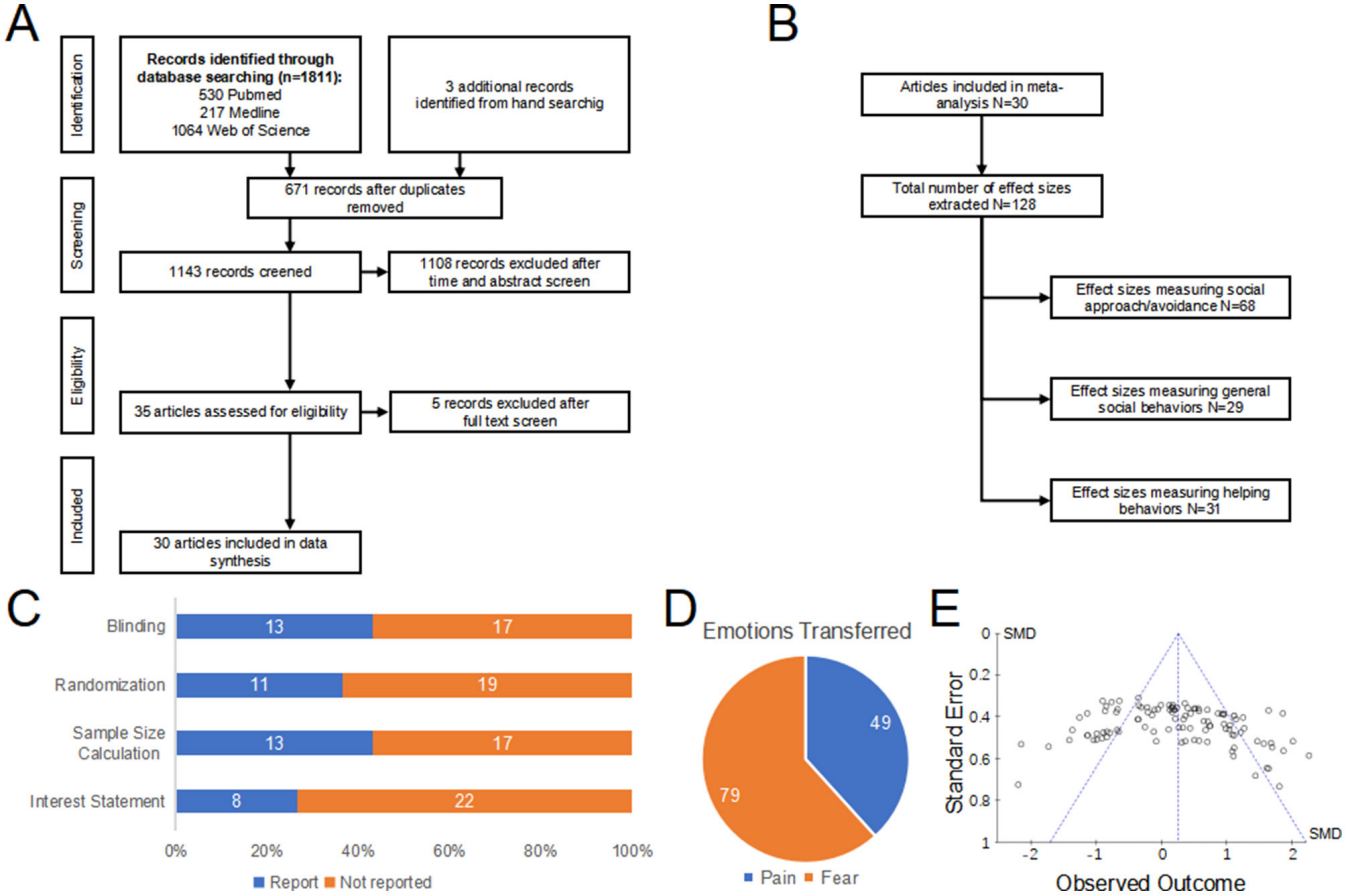
Descriptive output of the meta-analytic search. **(A)** Flow chart depicting the search strategy together with the number of articles excluded in each step. **(B)** Flow chart depicting the number of effect sizes and classification strategy used for analysis. **(C)** Quality control of the studies scrutinized. Cumulative bar plot shows the proportion of studies that reported blinding, randomization, sample size calculation and statement of conflict of interest in the text. Absence of such details in the article was classified as not reported for that given article. **(D)** Types and proportions of emotions transferred in the selected studies. Categories of emotions use the nomenclature proposed by Panksepp (2011). **(E)** Funnel plot of the selected studies illustrating potential publication bias, with effect size (*r*) as the observed outcome in the *x*-axis and standard error in the *y*-axis.

A comprehensive search of the rodent emotional contagion literature ultimately identified 30 studies (Figure 2A; Supplementary Table S4). Among the 128 behavioral effect sizes, a portion (*N* = 68) directly measured social approach/avoidance, another portion (*N* = 29) assessed general social behaviors, and the remaining part (*N* = 31) evaluated helping behaviors (Figure 2B).

Quality control assessments of all studies revealed less than optimal number of papers reporting on blinding (*N* = 13) and randomization procedures (*N* = 11), with an additional 13 of studies reporting sample size calculations (Figure 2C). Twenty-two of the studies did not provide any statement regarding conflicts of interest in their reports.

Each paper underwent meticulous scrutiny to extract experimental details pertinent to the process of emotional contagion. Typically, studies on rodent emotional contagion were characterized by one individual experiencing a specific emotional state, while another observes the emotional manifestation (Atsak et al., 2011). While the emotional manifestation was generally produced by a conspecific, in many instances, these emotional states were initially elicited in a separate stimulus environment before the animals were moved to the observation setting to continue expressing secondary emotional states (Shi et al., 2022).

In calculating each effect size, we characterized the type of emotion transferred to the subjects, thereby eliciting an emotional contagion response. We adhered to the classification of emotions proposed by Panksepp (2011), which differentiates between positive emotions (care, lust, and play) and negative emotions (aggression, pain, and fear). Utilizing this classification revealed a significant gap in research on the emotional contagion of positive emotions. The majority of experiments investigated emotional contagion through negative emotions (fear: *N* = 79, pain: *N* = 49; Figure 2D). For negative emotions, we divided the studies into two groups: (1) fear, and (2) pain. This meta-analysis focuses on these two categories due to their prevalence in the research.

Additionally, we did not detect any asymmetry in the funnel plot, which indicates that publication bias is not significant (Figure 2E). It was noteworthy that the upper portion of the funnel plot, representing studies with greater power, was densely filled. It must be emphasized that a strong publication bias has been observed in conferences and oral communications within the field of rodent emotion contagion. Caution should be taken in interpreting this analysis, as it had been proposed to be unreliable in certain instances (Lau et al., 2006).

### 3.2 Social contagions of pain and fear result in comparable levels of social approach vs. social avoidance

**Figure 3:**
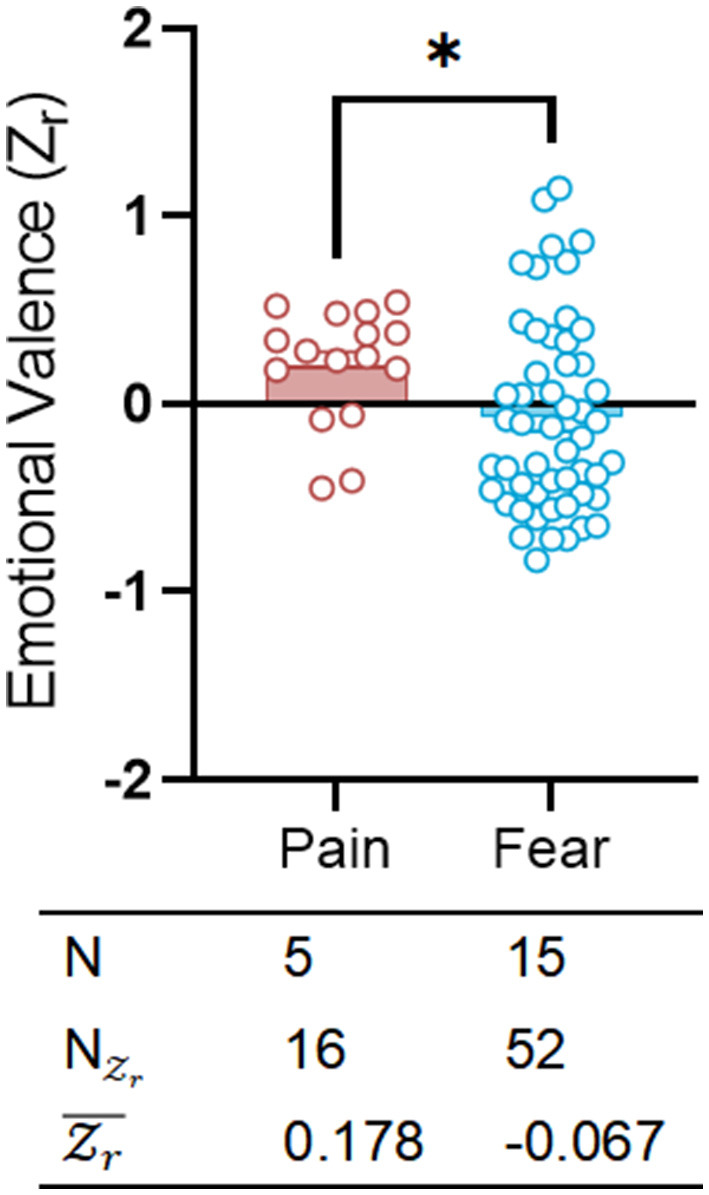
Exposure to painful and fearful catastrophe may result in different emotional valence. Scatter plot showing the distributions of emotional valence ($Z_{r}$) separately for pain (red) and fear (blue). The table on bottom shows the emotion category, species, sex, number of researches (*N*), number of emotional valence ($N_{Z_{r}}$) and mean emotional valence ($\bar{Z_{r}}$). Positive valence is associated with social approach/preference, while negative valence is associated with social avoidance. *p < 0.05.

We employed a three-chamber assay to assess the distribution of effect sizes across different emotional categories (Figure 3; Supplementary Table S5). Referencing significant distinctions in emotional responses (Panksepp, 2011), various conditions (fear and pain) exhibited diametrically polarized effect sizes in the three-chambers tests designed for emotional contagion [fear: $\bar{Z_{r}}$ = −0.067, CI: (−0.125 to −0.010); pain: $\bar{Z_{r}}$ = 0.178, CI: (0.068 to 0.288)]. Emotional contagion of fear revealed an instinctual tendency toward social avoidance in rodents, whereas emotional contagion of pain displayed a different pattern of proactive care, marked by social approach. Fear was more commonly observed than pain in three-chamber tests studying emotional contagion, with a greater prevalence of fear sets ($N_{Z_{r}}=$ 52) vs. pain sets ($N_{Z_{r}}=$ 16).

### 3.3 Witness of fearful catastrophe results in social avoidance in both rats and mice

**Figure 4:**
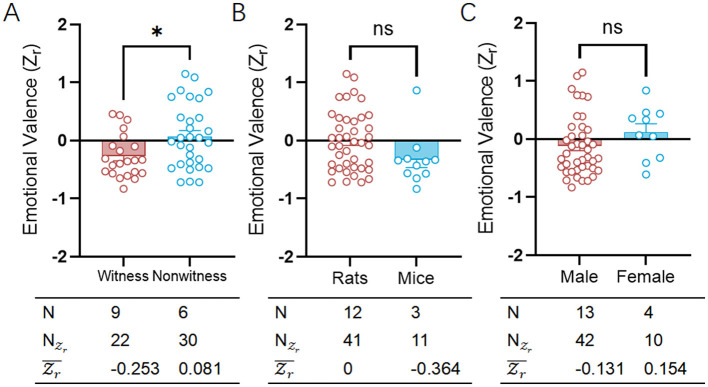
Comparative analysis of fear-related emotional valence between witness and nonwitness, rats and mice or male and female. **(A)** Scatter plot showing the distributions of emotional valence ($Z_{r}$) for witness (red) and nonwitness (blue) in the fear category. **(B)** Scatter plot showing the distributions of emotional valence ($Z_{r}$) for rats (red) and mice (blue) in the fear category. **(C)** Scatter plot showing the distributions of emotional valence ($Z_{r}$) for male (red) and female (blue) in the fear category. The table on bottom of each graph shows the emotion category, species, sexes, number of researches (*N*), number of emotional valence ($N_{Z_{r}}$) and mean emotional valence ($Z_{r}$). Positive valence is associated with social approach/preference, while negative valence is associated with social avoidance. ns, no significance; *p < 0.05.

In specific phobias, fear-related visual avoidance behaviors had been identified, underscoring the significant role of visual stimuli in fear responses (Tolin et al., 1999). Within test for emotional contagion of fear, both rats and mice showed a diversely high level of negative emotional valence (Figure 4A; Supplementary Table S5), indicating a trend toward social avoidance through witness [witness: $\bar{Z_{r}}$ = −0.253, CI: (−0.339 to −0.168); nonwitness: $\bar{Z_{r}}=$0.081, CI: (0.004 to 0.157)]. It's important to note that witness has been used similarly equal to nonwitness in these types of experiments to study the emotional contagion of fear (witness: $N_{Z_{r}}=$22, nonwitness: $N_{Z_{r}}=$30).

Despite variations in social structures and general sociability (Archer, 1973), both rats and mice demonstrated a comparably high negative emotional valence in a three-chamber test designed for emotional contagion of fear [rats: $\bar{Z_{r}}=$0.000, CI: (−0.063 to 0.063), mice: $\bar{Z_{r}}$ = −0.364, CI: (−0.497 to −0.232)], indicating a tendency toward social avoidance (Figure 4B; Supplementary Table S5). Notably, compared to mice, rats have been more frequently utilized in three-chambers experiments to investigate the emotional contagion of fear (rats: $N_{Z_{r}}$ = 41, mice: $N_{Z_{r}}$ = 11). Although there were sex differences in empathetic behaviors (Fang et al., 2024), male and female rodents alike demonstrated comparably significant negative emotional valence in a three-chambers assay tailored for assessing the emotional contagion of fear [male: $\bar{Z_{r}}$ = −0.131, CI: (−0.195 to −0.066), female: $\bar{Z_{r}}$ = 0.154, CI: (0.032 to 0.276)]. Sex-specific traits continued to reflect a consistent trend toward social avoidance, aligned with species-specific characteristics, without any noticeable differences (Figure 4C; Supplementary Table S5). Significantly, male was chosen more often than female for three-chambers tests exploring fear's emotional contagion, with a higher usage in male ($N_{Z_{r}}$ = 42) compared to female ($N_{Z_{r}}$ = 10).

### 3.4 Fear-induced social avoidance is enhanced with age in both rats and mice

**Figure 5:**
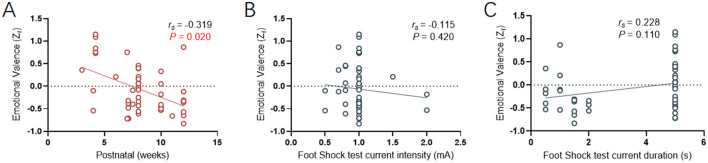
Correlative analysis between age or fear conditioning and emotional valence. **(A)** Linear regression showing the relationship between observers' age and emotional valence ($Z_{r}$). **(B, C)** Linear regression showing the relationship between current intensity (mA) or duration (s) and emotional valence ($Z_{r}$). Positive valence is associated with social approach/preference, while negative valence is associated with social avoidance.

The age of animals used in the fear contagion literature had a large range for both rats and mice [total range (1.7–14) weeks, $\bar{\chi }\;=$ 7.9]. A linear regression revealed a negative correlation (*r*_*s*_ = −0.319, *p* = 0.020) between the effect size of fear contagion and the age range (Figure 5A), suggesting that with increasing age, rodents are more prone to choose social avoidance behaviors when encountering fear contagion. This effect could reflect species specific age-related changes in additional factors such as animal cognition, experience and behavior (as animals get older, they recognize the dangers of the environment).

Speculating that fear conditioning apparatus tests could provide insights by observing mouse behavior related to fear contagion in relation to demonstrator mice receiving electric shocks, we indirectly evaluated the correlation between fear contagion's effect size and the intensity and duration of the electric current. The linear regression results revealed no correlation (Figures 5B, C) between the effect size of fear contagion and the settings of the two electric shock model parameters (Current intensity: *r*_*s*_ = −0.115, *p* = 0.420; Current duration: *r*_*s*_ = 0.228, *p* = 0.110). This finding inversely confirms our initial observations, indicating that with increasing age (acquired over time), there is an enhanced sensitivity to perceiving and responding to fear contagion.

### 3.5 Rats and mice exhibit spontaneous reduction in helping behaviors under fear contagion

In addition to analyzing and comparing the different trends between fear contagion and pain contagion in three-chamber test results, we also studied the differences between helping behaviors and general social behaviors between the two groups. This is because socially close contact behaviors often display a continuation of active approach (Suvilehto et al., 2015).

**Figure 6:**
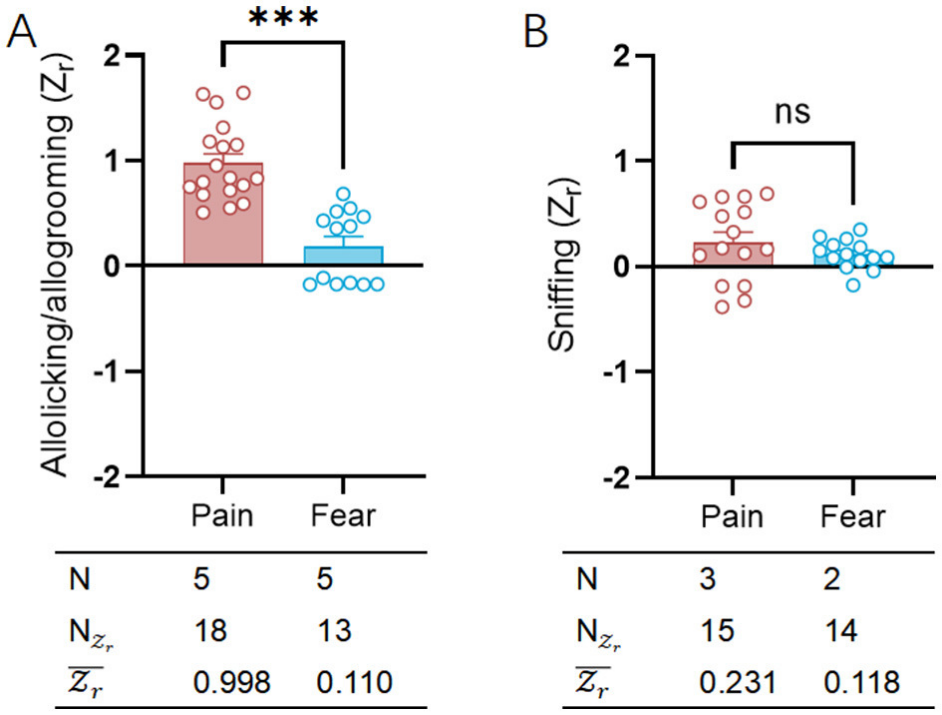
Comparative analysis of helping/caring and sniffing behavior between pain and fear contagion models. **(A)** Scatter plot showing the distributions of allolicking/allogrooming ($Z_{r}$) the observers engaged toward a demonstrator in pain (red) or in fear (blue). **(B)** Scatter plot showing the distributions of sniffing ($Z_{r}$) the observers engaged toward a demonstrator in pain (red) or in fear (blue). The table on bottom of each graph shows the emotion category, number of researches (*N*), number of emotional valence ($N_{Z_{r}}$) and mean emotional valence ($Z_{r}$). Positive valence is associated with increasing allolicking, allogrooming and sniffing behaviors, while negative valence is associated with decreasing allolicking, allogrooming and sniffing behaviors. ns, no significance; ***p < 0.001.

We utilized the helping behavior evaluation methods (Li et al., 2018) employed in previously published literature to assess the distribution of emotional valence across different emotional categories (Figure 6A; Supplementary Table S5). Referencing significant distinctions in emotional responses (Panksepp, 2011), the conditions (fear and pain) exhibited statistically significant effect sizes in helping behaviors designed for emotional contagion [fear: $\bar{Z_{r}}$ = 0.110, CI: (−0.001 to 0.222); pain: $\bar{Z_{r}}$ = 0.998, CI: (0.889 to 1.106)]. The emotional contagion of pain more frequently prompted helping behaviors, including caring and assisting actions, among rodent observers toward their conspecifics, a phenomenon that did not find a statistical counterpart in fear contagion. In the current study, records of helping behavior are less frequent in both fear (*N* = 5) and pain (*N* = 5) contagion. A larger sample size would likely improve the reliability and validity of these findings.

Under different conditions (fear and pain) designed for emotional contagion, general social behaviors did not (Figure 6B; Supplementary Table S5) show differing emotional valence [fear: $\bar{Z_{r}}$ = 0.118, CI: (0.021 to 0.222); pain: $\bar{Z_{r}}$ = 0.231, CI: (0.115 to 0.346)]. The social recognition and exploration ability exhibited by rodent observers toward their conspecifics did not vary with different intervention methods used on demonstrators. In the current study, there are fewer instances of general social behavior recorded for both fear contagion (*N* = 2) and pain contagion (*N* = 3).

## 4 Discussion

Our meta-analysis reveals distinct behavioral responses in rodents exposed to fear and pain contagion. Fear contagion primarily leads to social avoidance, which is intensified with age, whereas pain contagion promotes social approach and helping behaviors. These results do not differ by sex. These key findings highlight the complexity of emotional contagion and underscore the need to focus on the specific mechanisms underlying various emotional states.

Research has demonstrated that socially transmitted emotional information significantly influences the behavior of rodents when they encounter stressed conspecifics. The observed pattern is robust and consistent across sexes (Toyoshima et al., 2022). The exchange of emotional states between individuals and their actual experiences profoundly impacts their internal states and behaviors (Langford et al., 2006). Emotional information is conveyed through facial expressions, vocalizations, and odors, and is received through social interactions. This form of communication facilitates environmental adaptation and increases survival rates (Monfils and Agee, 2019). Detecting distress or fear in conspecifics is crucial for social animals, as such information often signals potential danger or threat (Ehret, 2005; Atsak et al., 2011). Fear is frequently acquired indirectly through group observation, with rodents exhibiting emotional sensitivity to the distress of their conspecifics and learning fear by observing others' suffering (Yehuda et al., 2015; Shi et al., 2022). Fear contagion induces social avoidance behaviors in rodents, which align with self-preservation instincts. Witnessing fear signals from conspecifics suggests potential danger, thus triggering avoidance behaviors to mitigate risk (Masuda and Aou, 2009; Jones et al., 2014). Observers engage in increased social exploratory behaviors, such as sniffing and grooming, likely to gather emotional state information from their peers and adjust their own behavior accordingly (Knapska et al., 2009). Brief social interactions with fear-conditioned conspecifics can enhance the observer's escape response and improve learning and memory of defensive behaviors. This indicates that fear is transmitted among conspecifics through social interactions, thereby enhancing defensive behaviors in potentially dangerous situations. Rodents are capable of recognizing fear expressions in conspecifics through visual signals and exhibit significant social avoidance behaviors. When exposed to images of conspecifics displaying fear expressions, rodents tend to avoid these images (Susskind et al., 2008; Nakashima et al., 2015). Interacting with conspecifics undergoing avoidance learning further strengthens rodents' avoidance behaviors. Witnessing fear signals from conspecifics elicits avoidance behaviors to reduce potential danger. Individuals with avoidance experience exhibit more pronounced avoidance behaviors compared to those without such experience, underscoring the importance of experience and age in fear contagion (Nakashima et al., 2015). Adult rodents show avoidance behaviors when exposed to stressed adult conspecifics but display more contact behaviors when exposed to juvenile conspecifics. This suggests that social stress signals from adults are perceived as danger cues, while those from juveniles are perceived as prosocial signals. With age, rodents tend more toward avoidance behaviors in fear contagion. Adult individuals may utilize vocal and chemical signals to assess the age and emotional state of conspecifics, resulting in adaptive behavioral responses (Rogers-Carter et al., 2018).

Pain contagion triggers social approach and increases helping behaviors, such as allogrooming and allolicking, reflecting empathy-driven actions to alleviate the distress of conspecifics. Observing conspecific pain activates neural circuits related to personal pain experiences, promoting prosocial behaviors, indicating that pain contagion not only elicits sympathy but also motivates comforting and helping (Chen, 2018). Familiar rodent observers are more likely to exhibit wound-licking and grooming behaviors toward others in pain, highlighting the importance of social familiarity in pain contagion and empathy-driven prosocial actions (Du et al., 2020). Pain contagion and consolation behaviors have been observed in rodents, with visual cues playing a crucial role in this transmission (Geng et al., 2020; Li et al., 2018). Past pain experiences trigger empathetic consolation behaviors in unfamiliar observing rodents, manifested by shorter latency to consolation, increased duration, and frequency of comforting behaviors (Luo et al., 2020). Pain contagion involves the activation of neural circuits associated with personal suffering. Rodents observing a conspecific in pain exhibit synchronized neural activity in the amygdala and anterior cingulate cortex (ACC), indicating these brain regions' significant roles in perceiving and responding to the distress of others (Panksepp and Lahvis, 2011). Activity in these areas correlates with the individual's own pain experiences, and activation of ACC neurons enhances social behaviors when observing conspecific pain, including increased helping actions (Paradiso et al., 2021). ACC neurons respond differently to the pain and general emotional stress of others, enabling rodents to distinguish between conspecific pain and emotional stress and to perform specific helping behaviors. Social licking differs from general social grooming, targeting injured areas in response to conspecific pain, whereas general grooming responds to emotional stress. Injured rodents reduce self-licking frequency when receiving social licking from conspecifics, suggesting that social licking not only comforts but also substitutes for self-care, effectively promoting recovery (Zhang et al., 2024). Voluntary physical contact enhances emotional contagion among rodents. When transparent barriers prevent physical contact, the effect of emotional contagion significantly diminishes, indicating the necessity of physical contact in emotional contagion and social bond formation (Lecker et al., 2021).

In experiments observing the contagion of fear among conspecifics, despite behavioral differences in emotional contagion between male and female rodents, these differences are not significant. This finding suggests that the phenomenon of emotional contagion in the context of fear transcends sex differences. Such insights prompt a reconsideration of traditional sex roles in animal behavior research, emphasizing the importance of contextual background when assessing animal emotional responses. Animals in their natural environments face various challenges and stressors, from obtaining food and avoiding predators to competing for territory and interacting with conspecifics (Brown and Orians, 1970; Grenier-Potvin et al., 2021). In the complexity and fluidity of these contexts, the mechanisms of animal emotional responses must be adaptive to cope with ever-changing environments (Scott, 2016; Novembre and Iannetti, 2021). Therefore, animals' responses to the emotional states of their conspecifics may be more influenced by external situational variables rather than solely by sex differences. This context-driven response may be an adaptation strategy finely tuned through evolution to optimize survival and reproductive success (Janicke and Chapuis, 2016; Janicke et al., 2022). In social living, quickly recognizing and responding to cues communicated by conspecifics is crucial for maintaining the stability of social groups (Yao et al., 2009; Ebina and Mizunami, 2020). This cross-sex emotional contagion allows social groups to more effectively coordinate behaviors, thereby enhancing their collective ability to cope with environmental challenges.

While our study provides valuable insights into the behavioral differences between fear and pain contagion in rodents, several limitations must be acknowledged. First, this meta-analysis primarily relies on previously published studies, which may introduce inherent biases related to study design, sample sizes, and reporting practices. Despite our efforts to mitigate these biases, the possibility of publication bias remains. Although our funnel plot analysis indicated no significant publication bias, the limited number of high-quality studies available for inclusion could still affect the generalizability of our findings. Secondly, the variability in experimental methodologies across studies posed challenges in standardizing effect sizes and drawing definitive conclusions. Differences in the protocols used to induce and measure fear and pain contagion, such as the type of stressor, duration of exposure, and specific behavioral assays, may have contributed to the observed heterogeneity. Future research should aim to develop standardized protocols to facilitate more consistent and comparable results. Furthermore, our study did not account for the potential influence of genetic background and strain differences among the rodent models used in the included studies. Genetic variability can significantly impact behavioral responses to emotional stimuli, and future studies should consider using genetically diverse populations to enhance the robustness of the findings. Additionally, relying solely on observational and behavioral measures may not fully capture the complexity of emotional contagion and empathy in rodents. While behaviors such as social approach, avoidance, and helping actions provide valuable insights, incorporating physiological and neural correlates of emotional contagion would offer a more comprehensive understanding. Techniques such as functional imaging, electrophysiology, and genetic manipulation could elucidate the underlying neural circuits and molecular pathways involved in these processes. Finally, our study focused on fear and pain contagion, but emotional contagion encompasses a broader range of emotions, including positive emotions such as joy and affection. The majority of existing research has concentrated on negative emotions due to their more apparent and measurable behavioral manifestations. Future studies should explore the contagion of positive emotions to provide a more balanced understanding of emotional contagion and its implications for social behavior and welfare.

In summary, our meta-analysis demonstrates that rodents exhibit distinct behavioral patterns under fear and pain contagion. Fear contagion primarily leads to social avoidance behaviors, whereas pain contagion is associated with increased social approach and helping behaviors. These findings highlight the importance of context and specific emotional stimuli in shaping social interactions. This research is significant for understanding rodent behavior and provides a neurobiological foundation for the evolutionary roots and social transfer of emotions in social animals. The results clearly suggest that the emotional valence induced by pain contagion aligns more closely with the mainstream concept of empathy. By addressing the limitations in our study and incorporating more diverse methodologies and populations, future research can further elucidate the mechanisms of emotional contagion and its role in empathy, ultimately contributing to the development of interventions aimed at enhancing social welfare.

## Data Availability

The datasets presented in this article are not readily available. Requests to access the datasets should be directed to ruidu_ibsp@163.com.
