# Supplementary material for: Social contagion of pain and fear results in opposite social behaviors in rodents: meta- analysis of experimental studies
Source: Front Behav Neurosci. 2024 Oct 29;18:1478456. doi: 10.3389/fnbeh.2024.1478456 (PMC11555602; doi:10.3389/fnbeh.2024.1478456)
Supplement: Supplementary file 1 [file Table_1.docx]

**Supplementary Table S1** Definition and description of animal behavior.

| Term | Explanation | Measurement |
| --- | --- | --- |
| Social behavior | An organized interaction between individuals of the same species, functionally linked to the spatial organization of the group and reproductive activities ([Barfield](https://pubmed.ncbi.nlm.nih.gov/?size=200&term=Barfield+RJ&cauthor_id=43971), 1979) | - |
| Prosocial behavior | A type of social behavior aimed at mutual benefit, voluntarily serving society (e.g., helping, sharing, donating, cooperating) from the perspective of others' interests, while adhering to social order and morality (de Waal and Preston, 2017) | - |
| Altruistic behavior | Behavior in which the subject selflessly provides help and comfort to a target in need, distress, or misfortune (de Waal and Preston, 2017) | - |
| Antisocial behavior | Hostile conduct expressed either covertly or overtly, typically manifested as actions that infringe upon the interests or safety of others ([Tielbeek](https://pubmed.ncbi.nlm.nih.gov/?size=200&term=Tielbeek+JJ&cauthor_id=36284158) et al., 2022) | - |
| Grooming | Scratching, grooming, or wiping, involving targeted limb movements and can occur without brain input in insects, amphibians, reptiles, and mammals (Berkowitz and Laurent, 1996) | - |
| Licking | Rhythmic oral motor movements involved in intraoral transport of fluid to the position for swallowing (Kaplan et al., 1995) | - |
| Allogrooming | Using mouth or teeth to clean another’s fur and skin, removing dirt, dead skin, parasites, or dried blood, and helping strengthen social bonds between individuals in the group (Sinha, 1998) | Time and bouts |
| Allolicking | Licking specifically targeting an injury site associated with empathy and care in response to the pain or distress of another (Du et al., 2024) | Time and bouts |
| Social approach | An individual actively initiating contact with others and attempting to engage in social interaction (Dodge et al., 1982) | Staying (percent, time and bouts) |
| Social avoidance | An active withdrawal behavior typically triggered by the perception of threat or unpleasant social experiences, manifesting in response to stress or anxiety-inducing situations (Gellner et al., 2024) | Staying (percent, time and bouts) |

Barfield, R.J. (1979). The hypothalamus and social behavior with special reference to the hormonal control of sexual behavior. *Poult Sci* 58, 61625-61632. doi: 10.3382/ps.0581625.

Berkowitz, A., and Laurent, G. (1996). Local control of leg movements and motor patterns during grooming in locusts. *Journal of neuroscience* 16, 8067–8078. doi: 10.1523/JNEUROSCI.16-24-08067.1996.

de Waal, F., and Preston, S. (2017). Mammalian empathy: behavioural manifestations and neural basis. *Nat Rev Neurosci* 18, 498-509. doi: 10.1038/nrn.2017.72.

Kaplan, J.M., Roitman, M.F., Grill, H.J. (1995). Ingestive taste reactivity as licking behavior. *Neurosci Biobehav Rev* 19, 89-98. doi:10.1016/0149-7634(94)00023-t.

Dodge, K.A., Coie, J.D., and Brakke, N.P. (1982). Behavior patterns of socially rejected and neglected preadolescents: the roles of social approach and aggression. *J Abnorm Child Psychol* 10, 389–409. doi: 10.1007/BF00912329.

Du, R., Lu, G., Luo, W., He, T., Li, C., Yu, Y., et al. (2024). Dyadic social interaction paradigm reveals selective role of ovarian estrogen in the caring behavior and socially transferred pain in female mice. *Neuropharmacology* 261, 110138. doi: 10.1016/j.neuropharm.2024.110138.

Gellner, A., Voelter, J., Schmidt, U., Beins, E., Stein, V., Philipsen, A., et al. (2021). Molecular and neurocircuitry mechanisms of social avoidance. *Cell Mol Life Sci* 78, 1163–1189. doi: 10.1007/s00018-020-03649-x.

Sinha A. (1998). Knowledge acquired and decisions made: triadic interactions during allogrooming in wild bonnet macaques, Macaca radiata. *Philos Trans R Soc Lond B Biol Sci* 353, 619–631. doi: 10.1098/rstb.1998.0230.

Tielbeek, J.J., Uffelmann, E., Williams, B.S., Colodro-Conde, L., Gagnon, É., Mallard, T.T., et al. (2022). Uncovering the genetic architecture of broad antisocial behavior through a genome-wide association study meta-analysis. *Molecular psychiatry* 27, 4453–4463. doi: 10.1038/s41380-022-01793-3.
