# Supplementary material for: Social contagion of pain and fear results in opposite social behaviors in rodents: meta- analysis of experimental studies
Source: Front Behav Neurosci. 2024 Oct 29;18:1478456. doi: 10.3389/fnbeh.2024.1478456 (PMC11555602; doi:10.3389/fnbeh.2024.1478456)
Supplement: Supplementary file 2 [file Table_2.docx]

**Supplementary Table S2** Encoded variables, data type and possible outcomes

| Code variable | Data type | Possible outcome |
| --- | --- | --- |
| Sex | Categorical | Male or Female |
| Emotion transfer | Categorical | Fear, Pain (Panksepp, 2011) |
| Witness/Nonwitness | Categorical | Yes or no |
| Three-chamber test | Categorical | Yes (Analogy) or no |
| General social behavior | Categorical | Yes or no |
| Helping behavior | Categorical | Yes or no |

Panksepp, J. (2011). The basic emotional circuits of mammalian brains: do animals have affective lives? *Neurosci Biobehav Rev* 35, 1791-1804. doi: 10.1016/j.neubiorev.2011.08.003.
