# Supplementary material for: Social contagion of pain and fear results in opposite social behaviors in rodents: meta- analysis of experimental studies
Source: Front Behav Neurosci. 2024 Oct 29;18:1478456. doi: 10.3389/fnbeh.2024.1478456 (PMC11555602; doi:10.3389/fnbeh.2024.1478456)
Supplement: Supplementary file 3 [file Table_3.DOCX]

**Supplementary Table S3** Descriptions of encoded characteristics that were used to minimize risk of bias quantification

| Encoded characteristic | Description |
| --- | --- |
| Blinding | Blinding was considered present when the experimenter was blinded to the experimental conditions during the analysis. |
| Randomization | Randomization was counted as present when it was used as a procedure at any point during the study and assumed missing otherwise |
| Sample Size Calculation | Sample size calculation was counted when explicitly mentioned in the text and assumed missing otherwise |
| Conflict of Interest Statement | No conflict of interest was reported when explicitly stated in the article. |
