# Supplementary material for: Social contagion of pain and fear results in opposite social behaviors in rodents: meta- analysis of experimental studies
Source: Front Behav Neurosci. 2024 Oct 29;18:1478456. doi: 10.3389/fnbeh.2024.1478456 (PMC11555602; doi:10.3389/fnbeh.2024.1478456)
Supplement: Supplementary file 4 [file Table_4.docx]

**Supplementary Table S4** List of included studies, together with categories of emotional transfer, number of effect sizes (ES) for three-chamber test (social approach/avoidance), general social behavior and helping behavior

|  | Study & Year | Emotional  transfer | ES-social approach/avoidance | ES-general  social behavior | ES-  helping behavior |
| --- | --- | --- | --- | --- | --- |
| 1 | Armario et al., 1983 | Fear | 1 | 0 | 0 |
| 2 | Atsak et al., 2011 | Fear | 4 | 0 | 0 |
| 3 | Carrillo et al., 2015 | Fear | 1 | 0 | 0 |
| 4 | Kiyokawa et al., 2019 | Fear | 2 | 0 | 0 |
| 5 | Jones et al., 2014 | Fear | 1 | 0 | 0 |
| 6 | Knapska et al., 2009 | Fear | 0 | 2 | 1 |
| 7 | Nakashima et al., 2015 | Fear | 2 | 0 | 0 |
| 8 | Pisansky et al., 2017 | Fear | 6 | 0 | 0 |
| 9 | Rogers-Carter and Djerdjaj et al., 2018 | Fear | 6 | 0 | 0 |
| 10 | Rogers-Carter and Varela et al., 2018 | Fear | 6 | 0 | 0 |
| 11 | Shi et al., 2022 | Fear | 3 | 0 | 0 |
| 12 | Toyoshima et al., 2021 | Fear | 2 | 0 | 0 |
| 13 | Toyoshima et al., 2022 | Fear | 10 | 0 | 0 |
| 14 | Yusufishaq and Rosenkranz, 2013 | Fear | 2 | 0 | 0 |
| 15 | Burkett et al., 2016 | Fear | 0 | 0 | 2 |
| 16 | Mikosz et al., 2016 | Fear | 0 | 12 | 6 |
| 17 | Wu et al., 2021 | Fear | 2 | 0 | 2 |
| 18 | Phillips et al., 2023 | Fear | 0 | 0 | 2 |
| 19 | Rieger et al., 2023 | Fear | 4 | 0 | 0 |
| 20 | Gioiosa et al., 2009 | Pain | 4 | 0 | 0 |
| 21 | Langford et al., 2010 | Pain | 5 | 0 | 0 |
| 22 | Ueno et al., 2018 | Pain | 4 | 0 | 0 |
| 23 | Watanabe, 2012 | Pain | 1 | 0 | 0 |
| 24 | Du et al., 2020 | Pain | 0 | 12 | 8 |
| 25 | Li et al., 2018 | Pain | 0 | 0 | 2 |
| 26 | Luo et al., 2020 | Pain | 0 | 0 | 2 |
| 27 | Lu et al., 2018 | Pain | 0 | 0 | 2 |
| 28 | Smith et al., 2021 | Pain | 0 | 1 | 0 |
| 29 | Zhang et al., 2024 | Pain | 0 | 2 | 4 |
| 30 | Li et al., 2024 | Pain | 2 | 0 | 0 |

Armario, A., Ortiz, R., and Balasch, J. (1983). Corticoadrenal and behavioral response to open field in pairs of male rats either familiar or non-familiar to each other. *Experientia* 39, 1316-1317. doi: 10.1007/BF01990391.

Atsak, P., Orre, M., Bakker, P., Cerliani, L., Roozendaal, B., Gazzola, V., et al. (2011). Experience modulates vicarious freezing in rats: a model for empathy. *Plos One* 6, e21855. doi: 10.1371/journal.pone.0021855.

Burkett, J., Andari, E., Johnson, Z., Curry, D., de Waal, F., and Young, L. (2016). Oxytocin-dependent consolation behavior in rodents. *Science* 351, 375-378. doi: 10.1126/science.aac4785.

Carrillo, M., Migliorati, F., Bruls, R., Han, Y., Heinemans, M., Pruis, I., et al. (2015). Repeated witnessing of conspecifics in pain: effects on emotional contagion. *Plos One* 10, e136979. doi: 10.1371/journal.pone.0136979.

Du, R., Luo, W., Geng, K., Li, C., Yu, Y., Wei, N., et al. (2020). Empathic contagious pain and consolation in laboratory rodents: species and sex comparisons. *Neurosci Bull* 36, 649-653. doi: 10.1007/s12264-020-00465-y.

Gioiosa, L., Chiarotti, F., Alleva, E., and Laviola, G. (2009). A trouble shared is a trouble halved: social context and status affect pain in mouse dyads. *Plos One* 4, e4143. doi: 10.1371/journal.pone.0004143.

Jones, C., Riha, P., Gore, A., and Monfils, M. (2014). Social transmission of pavlovian fear: fear-conditioning by-proxy in related female rats. *Anim Cogn* 17, 827-834. doi: 10.1007/s10071-013-0711-2.

Kiyokawa, Y., Li, Y., and Takeuchi, Y. (2019). A dyad shows mutual changes during social buffering of conditioned fear responses in male rats. *Behav Brain Res* 366, 45-55. doi: 10.1016/j.bbr.2019.03.024.

Knapska, E., Mikosz, M., Werka, T., and Maren, S. (2009). Social modulation of learning in rats. *Learn Mem* 17, 35-42. doi: 10.1101/lm.1670910.

Langford, D., Tuttle, A., Brown, K., Deschenes, S., Fischer, D., Mutso, A., et al. (2010). Social approach to pain in laboratory mice. *Soc Neurosci* 5, 163-170. doi: 10.1080/17470910903216609.

Li, C., Yu, Y., He, T., Wang, R., Geng, K., Du, R., et al. (2018). Validating rat model of empathy for pain: effects of pain expressions in social partners. *Front Behav Neurosci* 12, 242. doi: 10.3389/fnbeh.2018.00242.

Li, J., Qin, Y., Zhong, Z., Meng, L., Huang, L., and Li, B. (2024). Pain experience reduces social avoidance to others in pain: a c-fos-based functional connectivity network study in mice. *Cereb Cortex* 34, bhae207. doi: 10.1093/cercor/bhae207.

Lu, Y., Ren, B., Ling, B., Zhang, J., Xu, C., and Li, Z. (2018). Social interaction with a cagemate in pain increases allogrooming and induces pain hypersensitivity in the observer rats. *Neurosci Lett* 662, 385-388. doi: 10.1016/j.neulet.2017.10.063.

Luo, W., Li, C., Geng, K., Wang, X., Du, R., Yu, Y., et al. (2020). The similar past pain experience evokes both observational contagious pain and consolation in stranger rat observers. *Neurosci Lett* 722, 134840. doi: 10.1016/j.neulet.2020.134840.

Mikosz, M., Nowak, A., Werka, T., and Knapska, E. (2016). Sex differences in social modulation of learning in rats. *Sci Rep* 5, 18114. doi: 10.1038/srep18114.

Nakashima, S., Ukezono, M., Nishida, H., Sudo, R., and Takano, Y. (2015). Receiving of emotional signal of pain from conspecifics in laboratory rats. *R Soc Open Sci* 2, 140381. doi: 10.1098/rsos.140381.

Phillips, H., Dai, H., Choi, S., Jansen-West, K., Zajicek, A., Daly, L., et al. (2023). Dorsomedial prefrontal hypoexcitability underlies lost empathy in frontotemporal dementia. *Neuron* 111, 797-806. doi: 10.1016/j.neuron.2022.12.027.

Pisansky, M., Hanson, L., Gottesman, I., and Gewirtz, J. (2017). Oxytocin enhances observational fear in mice. *Nat Commun* 8, 2102. doi: 10.1038/s41467-017-02279-5.

Rieger, N., Ng, A., Lee, S., Brady, B., and Christianson, J. (2023). Maternal immune activation alters social affective behavior and sensitivity to corticotropin releasing factor in male but not female rats. *Horm Behav* 149, 105313. doi: 10.1016/j.yhbeh.2023.105313.

Rogers-Carter, M., Djerdjaj, A., Culp, A., Elbaz, J., and Christianson, J. (2018). Familiarity modulates social approach toward stressed conspecifics in female rats. *Plos One* 13, e200971. doi: 10.1371/journal.pone.0200971.

Rogers-Carter, M., Varela, J., Gribbons, K., Pierce, A., McGoey, M., Ritchey, M., et al. (2018). Insular cortex mediates approach and avoidance responses to social affective stimuli. *Nat Neurosci* 21, 404-414. doi: 10.1038/s41593-018-0071-y.

Shi, T., Feng, S., Shi, W., Fu, Y., and Zhou, W. (2022). A modified mouse model for observational fear learning and the influence of social hierarchy. *Front Behav Neurosci* 16, 941288. doi: 10.3389/fnbeh.2022.941288.

Smith, M., Asada, N., and Malenka, R. (2021). Anterior cingulate inputs to nucleus accumbens control the social transfer of pain and analgesia. *Science* 371, 153-159. doi: 10.1126/science.abe3040.

Toyoshima, M., Mitsui, K., and Yamada, K. (2021). Prior stress experience modulates social preference for stressed conspecifics in male rats. *Neurosci Lett* 765, 136253. doi: 10.1016/j.neulet.2021.136253.

Toyoshima, M., Okuda, E., Hasegawa, N., Kaseda, K., and Yamada, K. (2022). Socially transferred stress experience modulates social affective behaviors in rats. *Neuroscience* 502, 68-76. doi: 10.1016/j.neuroscience.2022.08.022.

Ueno, H., Suemitsu, S., Murakami, S., Kitamura, N., Wani, K., Okamoto, et al. (2018). Empathic behavior according to the state of others in mice. *Brain Behav* 8, e986. doi: 10.1002/brb3.986.

Watanabe, S. (2012). Distress of mice induces approach behavior but has an aversive property for conspecifics. *Behav Processes* 90, 167-173. doi: 10.1016/j.beproc.2012.01.001.

Wu, Y., Dang, J., Kingsbury, L., Zhang, M., Sun, F., Hu, R., et al. (2021). Neural control of affiliative touch in prosocial interaction. *Nature* 599, 262-267. doi: 10.1038/s41586-021-03962-w.

Yusufishaq, S., and Rosenkranz, J. (2013). Post-weaning social isolation impairs observational fear conditioning. *Behav Brain Res* 242, 142-149. doi: 10.1016/j.bbr.2012.12.050.

Zhang, M., Wu, Y., Jiang, M., and Hong, W. (2024). Cortical regulation of helping behaviour towards others in pain. *Nature* 626, 136-144. doi: 10.1038/s41586-023-06973-x.
