# Supplementary material for: Social contagion of pain and fear results in opposite social behaviors in rodents: meta- analysis of experimental studies
Source: Front Behav Neurosci. 2024 Oct 29;18:1478456. doi: 10.3389/fnbeh.2024.1478456 (PMC11555602; doi:10.3389/fnbeh.2024.1478456)
Supplement: Supplementary file 5 [file Table_5.DOCX]

**Supplementary Table S5** Values of the mean effect size ($\bar{\mathcal{Z}_{r}}$) with confidence interval ($CI$), z score, heterogeneity as measured by $I^{2}$ and sample size ($N_{\mathcal{Z}_{r}}$) calculated in terms of modulators, levels and sublevels

| Modulator | Levels | Sublevels | $\bar{\mathcal{Z}_{r}} (CI)$ | z | $I^{2}$ % | $N_{\mathcal{Z}_{r}}$ |
| --- | --- | --- | --- | --- | --- | --- |
| Three chamber test | Pain | - | 0.178  (0.068-0.288) | 3.194 | 51.8 | 16 |
|  | Fear | - | -0.067  (-0.125--0.010) | -2.328 | 81.1 | 52 |
|  |  | Witness | -0.253  (-0.339--0.168) | -5.801 | 70.4 | 22 |
|  |  | Non-witness | 0.081  (0.004-0.157) | 2.069 | 82.6 | 30 |
|  |  | Rat | 0.000  (-0.063-0.063) | 0.003 | 81.5 | 41 |
|  |  | Mice | -0.364  (-0.497--0.232) | -5.395 | 67.7 | 11 |
|  |  | Male | -0.131  (-0.195--0.066) | -3.961 | 79.6 | 42 |
|  |  | Female | 0.154  (0.032-0.276) | 2.487 | 83.1 | 10 |
| Helping behavior | Pain | - | 0.998  (0.889-1.106) | 18.025 | 62.2 | 18 |
|  | Fear | - | 0.110  (-0.001-0.222) | 1.939 | 63 | 13 |
| Geneal social behavior | Pain | - | 0.231  (0.115-0.346) | 3.916 | 61 | 15 |
|  | Fear | - | 0.121  (0.020-0.221) | 2.362 | 0 | 14 |
