# Supplementary material for: Social contagion of pain and fear results in opposite social behaviors in rodents: meta- analysis of experimental studies
Source: Front Behav Neurosci. 2024 Oct 29;18:1478456. doi: 10.3389/fnbeh.2024.1478456 (PMC11555602; doi:10.3389/fnbeh.2024.1478456)
Supplement: Supplementary file 6 [file Table_6.doc]

**Supplementary Table S6** Statistical analyses for corresponding figures in terms of the number of normalized effect sizes, normality test, equal variance test, statistical methods and *p* or *t(U)* values

| Figure number | No of animals | Normality test | Equal variance test | Statistic method | *p* value | *t(U)* value |
| --- | --- | --- | --- | --- | --- | --- |
| 3 | n=16 pain  n=52 fear | failed |  | two-tailed M-W *U* test | *p* = 0.015 | *U =* 584.000 |
| 4A | n=22 witness  n=30 nonwitness | passed | passed | two-tailed M-W *U* test | *p* = 0.030 | *U =* 212.500 |
|  |  |  |  | two-sample *t*-test | *p* = 0.016 | *t* = 2.497 |
| 4B | n=41 rats  n=11 mice | failed |  | two-tailed M-W *U* test | *p* = 0.050 | *U =* 138.000 |
| 4C | n=42 male  n=10 female | failed |  | two-tailed M-W *U* test | *p* = 0.102 | *U =* 280.500 |
| 6A | n=18 pain  n=13 fear | failed |  | two-tailed M-W *U* test | *p* < 0.001 | *U =* 6.000 |
| 6B | n=15 pain  n=14 fear | passed | failed | two-tailed M-W *U* test | *p* = 0.290 | *U =* 80.000 |

Notes: The non-parametric statistical analysis method was selected due to failure by either of normality test or equal variance test. *p* < 0.05 was considered as statistically significant, M-W, Mann-Whitney.
