# Supplementary material for: Social contagion of pain and fear results in opposite social behaviors in rodents: meta- analysis of experimental studies
Source: Front Behav Neurosci. 2024 Oct 29;18:1478456. doi: 10.3389/fnbeh.2024.1478456 (PMC11555602; doi:10.3389/fnbeh.2024.1478456)
Supplement: Supplementary file 7 [file Table_7.doc]

**Supplementary Table S7** Statistical analyses for corresponding figures in terms of the number of normalized effect sizes, normality test, statistic method, *p* and *r_s_* values, 95 % confidence interval (95 % CI), and coefficient of determination (*R^2^*)

| Figure number | n | Normality test | Statistic method | *p* value | Coefficient correlation | 95 % confidence interval | Coefficient of determination |
| --- | --- | --- | --- | --- | --- | --- | --- |
| 5A | 52 | failed | Spearman rank correlation | *p* = 0.020 | *r_s_* = -0.319 | (-0.550, -0.042) | *R^2^* = 0.101 |
| 5B | 51 | failed | Spearman rank correlation | *p* = 0.420 | *r_s_* = -0.115 | (-0.386, 0.173) | *R^2^* = 0.013 |
| 5C | 51 | failed | Spearman rank correlation | *p* = 0.110 | *r_s_* = 0.228 | (-0.058, 0.480) | *R^2^* = 0.051 |

Notes: *R^2^* meant squared of *r_s_*.
